# Ionizable Lipids with Triazole Moiety from Click Reaction for LNP-Based mRNA Delivery

**DOI:** 10.3390/molecules28104046

**Published:** 2023-05-12

**Authors:** Yixiang Wang, Xiao Si, Yi Feng, Dan Feng, Xiaoyu Xu, Yan Zhang

**Affiliations:** 1 School of Chemistry and Chemical Engineering, Chemistry and Biomedicine Innovation Center (ChemBIC), Jiangsu Key Laboratory of Advanced Organic Materials, State Key Laboratory of Analytical Chemistry for Life Sciences, Nanjing University, Nanjing 210023, China; 2 Nanjing Vazyme Biotechnology Company, Nanjing 210034, China

**Keywords:** ionizable lipids, lipid nanoparticles, click reaction, mRNA delivery

## Abstract

Ionizable lipid-containing lipid nanoparticles (LNPs) as a non-viral vector with good safety and potency have been considered as an ideal delivery system for gene therapy. The screening of ionizable lipid libraries with common features but diverse structures holds the promise of finding new candidates for LNPs to deliver different nucleic acid drugs such as messenger RNAs (mRNAs). Chemical strategies for the facile construction of ionizable lipid libraries with diverse structure are in high demand. Here, we report on the ionizable lipids containing the triazole moiety prepared by the copper-catalyzed alkyne–azide click reaction (CuAAC). We demonstrated that these lipids served well as the major component of LNPs, in order to encapsulate mRNA using luciferase mRNA as the model system. Thus, this study shows the potential of click chemistry in the preparation of lipid libraries for LNP assembly and mRNA delivery.

## 1. Introduction

Nucleic acid drugs such as messenger RNA (mRNA) can achieve long-lasting or even curative therapeutic effects through modulating gene expression [1]. However, due to the large size, hydrophilicity, negative charge and nuclease susceptibility, it is still challenging to deliver nucleic acid drugs into target cells. To address this challenge, lipid nanoparticles (LNPs) capable of improving the cellular permeability and cytosolic delivery capacity of nucleic acid drugs have been developed, representing the most clinically advanced delivery platform [2]. The global outbreak of the coronavirus disease (COVID-19) pandemic and the rapid approval of two mRNA vaccines have brought the LNPs that lie behind the success of nucleic acid drugs into the spotlight of both the scientific community and the general public [3]. The development of high-performance LNPs to further improve delivery efficiency and promote the clinical translation of diverse nucleic acid drugs is therefore of current research interest [4].

The four-component LNP became a leading non-viral vector for mRNA delivery after its use by Pfizer and Moderna in the development of COVID-19 vaccines [5]. Except for the ionizable lipids in FDA-approved LNP formulations, such as **SM-102** and **DLin-MC3-DMA**, various analogues or derivatives of these known lipids have been prepared and tested for their potential applications in LNP-based mRNA delivery [6]. Some of the chemical modifications on ionizable lipids were reported to endow LNPs with special properties. For example, the introduction of unsaturation into the structure of ionizable lipids greatly improved the mRNA transfection in vivo [7], and the incorporation of piperazine into ionizable lipids preferentially delivered mRNA into immune cells in vivo [8].

For a systematic investigation on the structure–activity relationship of the ionizable lipids in LNPs, different chemical strategies to construct the different libraries of these molecules have been developed in recent years. Michael addition has been applied, in order to prepare the lipid compound libraries by reacting acrylamide tails with different amines [9]. Epoxide ring-opening reactions [10] are used for alkylation modifications to synthesize different anionic lipids. Enzyme-assisted ligation reactions have also been applied to elevate the efficiency of library construction [11]. The azide–alkyne click reaction, catalyzed by a copper catalyst or promoted by an internal strain, has found diverse applications both in chemical–biological studies, and in fast ligation applications, since it features fast reaction rates and high yields of cycloaddition products under mild conditions compatible with bio-active molecules [12,13]. The cycloaddition product of click reactions contains triazole moieties, which is highly compatible with biological systems. We envision that click reactions should be able to serve as a facile method to construct compound libraries of ionizable lipids containing triazole moieties. However, whether the triazole-containing lipid molecules are able to serve as the major component of LNPs for mRNA delivery is unknown. Here, we report our work on the preparation of triazole-containing ionizable lipids based on the copper-catalyzed click reaction (CuAAC) and provide an evaluation of the potential of these lipids in the assembly of LNPs for mRNA delivery.

## 2. Results and Discussion

### 2.1. Synthesis of Triazole-Containing Ionizable Lipids

**Scheme 1:**
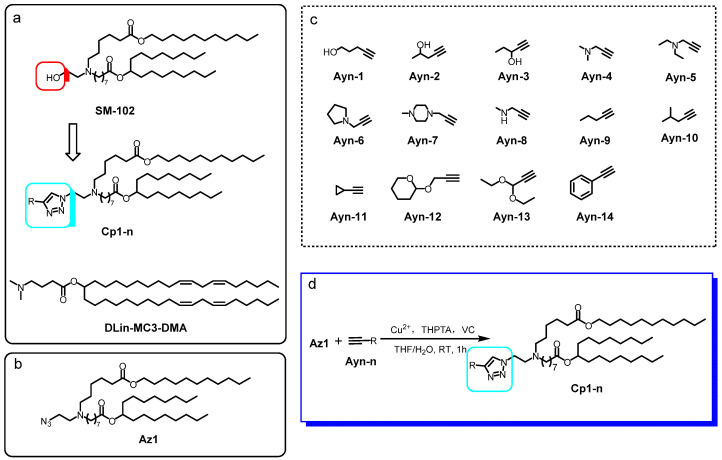
Chemical structure of (**a**) framework of the triazole-containing ionizable lipids with structural similarity to **SM-102** and **DLin-MC3-DMA**; (**b**) the azide substrate **Az1**; (**c**) different alkynes **Ayn-1~15**. (**d**) Reaction scheme between **Az1** and **Ayn-n** to give **Cp1-n**.

Based on the chemical structure of **SM-102**, which is the major component of the FDA-approved LNP for mRNA vaccines, we designed the framework of **Cp1-n**, which is made up of a series of ionizable lipids containing triazole moiety (Scheme 1a). The lipid tail in **Cp1-n** was the same as that in **SM-102**, while the hydroxyl group in **SM-102** was replaced by a series of triazoles with different substitution groups on the 4-position of the triazole structure.

We first prepared the ionizable lipid **Az1** with the azide handle at the reaction scale (Scheme 1b), following the protocol described in the Supporting Information, in order to obtain grams of **Az1.** Then, **Az1** was allowed to react with a variety of terminal alkynes under the CuAAC standard condition [14] (Scheme 1d). As shown in Scheme 1c, the **Ayn-1~15** with terminal alkynes chosen to react with **Az1** were all readily available from a commercial source. The substitution groups on the other end of the terminal alkyne molecules included alkyl, phenyl, alkylhydroxyl, alkylamino and alkyloxyl groups. With the diversity of the substitution groups in the terminal alkyne substrates, we were able to obtain the ionizable molecules with common frameworks and diversity on the substitution groups on the triazole ring linked with the lipid tail. The CuAAC reaction between **Az1** and **Ayn-n** was performed using tris(3-hydroxypropyltriazolylmethyl) amine (THPTA) and copper (I) generated from copper sulfate, together with vitamin C (VC). During the reaction, the azide group in **Az1** served as the 1, 3-dipole and underwent a [3+2] cycloaddition reaction with terminal alkynes in **Ayn-1~15**, in order to generate 1, 2, 3-triazoles **Cp1-n**. The **Cp1-n** were then purified and yields of over 80% were obtained, indicating the advantages of CuAAC for the efficient construction of ionizable lipid libraries.

### 2.2. Biocompatibility of Triazole-Containing Ionizable Lipids

To determine whether the triazole introduced into ionizable lipids was biocompatible, we measured cell viability using the Cell Counting Kit-8 (CCK-8) assay. As shown in Figure S1, the relative viabilities of human ovarian cancer HeLa cells treated with **Cp1-n** were over 80% at concentrations up to 100 μM. This result thus indicates a good biosafety that these triazole-containing ionizable lipids may exist, showing that they are valuable for further research in the preparation of LNPs and delivery of messenger RNA.

### 2.3. Preparation and Characterization of LNPs with Cp1-n as the Ionizable Lipids

**Figure 1:**
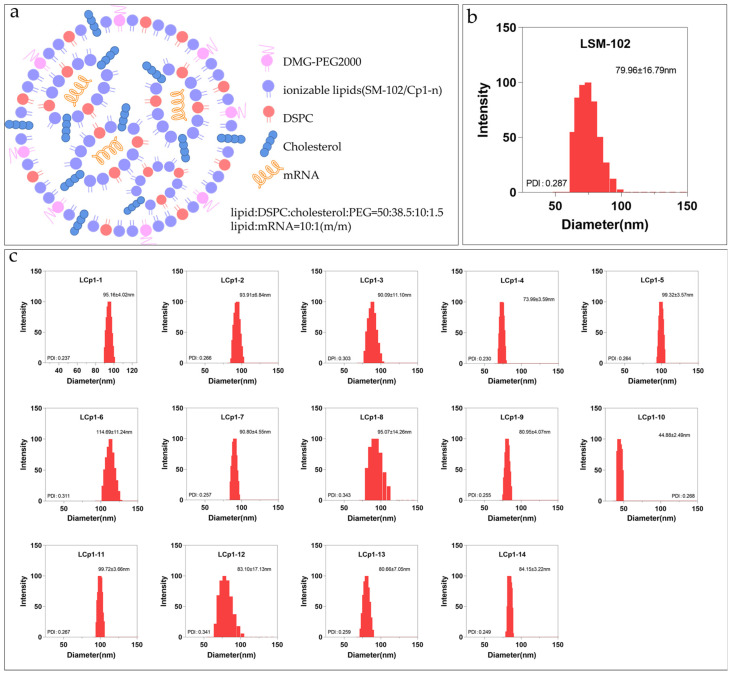
(**a**) Schematic illustration of the LNP formulations. (**b**,**c**) DLS analysis of LNPs formed of **SM-102** or **Cp1-n**.

According to the formula for the FDA-approved LNP for the mRNA vaccine against COVID-19 by Moderna [15], the LNP was prepared with the ionizable lipid **SM-102**, the helper lipid distearoylphosphatidylcholine (DSPC), cholesterol and the PEGylated lipid at a molar ratio of 50:10:38.5:1.5, respectively. We prepared **Cp1-n**-based LNPs using a similar cocktail protocol, replacing **SM-102** with **Cp1-n** (Figure 1a) [16]. Briefly, the ionizable lipid **Cp1-n** (or **SM-102**), DSPC, cholesterol and the PEGylated lipid were mixed, followed by the addition of luciferase mRNA at a 20:1 mass ratio of ionizable lipids to mRNA. These LNPs were then obtained and characterized using dynamic light scattering (DLS) analysis. As shown in Figure 1b, LNPs containing **SM-102** had a diameter of 79.96 ± 16.79 nm. Similarly, LNPs containing **Cp1-n** all formed nanoparticles, with sizes in the range of ~45 nm to ~113 nm (Figure 1c). For example, **Cp1-4**-based LNP was uniformly distributed, with a mean size of 73.99 ± 3.59 nm. These results thus indicate that the triazole-containing ionizable lipids **Cp1-n** can readily self-assemble with other components into LNPs. Specifically, the particle size of **Cp1-10** is only around 45 nm. This difference is due to the inherent defect of the liposome extruder. In fact, many studies reported that the size of LNPs is related to the mixing flow rate [17,18,19]. In the case of a fast flow rate, LNPs will show a smaller size; while the mixing flow rate cannot be controlled by the liposome extruder, the size of the LNP can be controlled as uniformly as possible and kept below 100 nm by using a polycarbonate membrane. Moreover, all LNPs formed of **Cp1-n** had positive zeta potentials ranging from +8.64 mV to +25.41 mV (Table 1), suggesting that they can readily cross negatively charged cell membranes.

### 2.4. Encapsulation Rates of mRNA in the Cp1-n-Based LNPs

To determine whether the triazole in ionizable lipids would affect the encapsulation of mRNA into LNPs, we quantified the encapsulation rates using the RiboGreen fluorescence probe method [20]. The principle behind using a RiboGreen reagent kit to measure encapsulation efficiency is that when RiboGreen binds to mRNA, fluorescence is generated, resulting in the detection of unencapsulated mRNA; after destroying the LNP structure with Triton, the total mRNA can be measured, and the difference is the encapsulated mRNA. As shown in Table 2, LNPs formed of **SM-102** had an encapsulation capability of 89.98%, and **Cp1-n**-based LNPs had comparable encapsulation rates ranging from 84.10% to 91.64%. These results indicate that the presence of triazole linkers would not affect the binding of ionized lipids to mRNA and LNP assembly.

### 2.5. mRNA Delivery by the Cp1-n-Based LNPs

**Figure 2:**
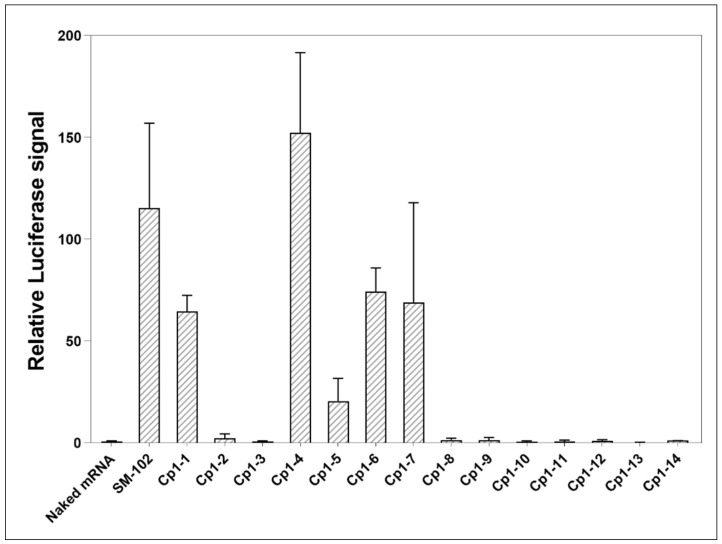
Relative luciferase signals from HeLa cells treated with naked mRNA, mRNA-encapsulated LNPs formed of **SM-102** or **Cp1-n** for 24 h. Data are shown as mean ± SEM (*n* = 3).

Next, we measured the transfection efficacies of **Cp1-n**-based LNPs. Briefly, HeLa cells were treated with **SM-102**- or **Cp1-n**-based LNPs containing luciferase mRNA, followed by the quantification of bioluminescence signals from proteins expressed by luciferase mRNA using luciferin substrates. As shown in Figure 2, LNPs formed of **Cp1-n** had variable mRNA delivery efficiencies. Specifically, compared to LNPs formed of **SM-102**, LNPs formed of **Cp1-1**, **Cp1-4**, **Cp1-5**, **Cp1-6** and **Cp1-7** had 0.60, 1.32, 0.18, 0.64 and 0.60-fold bioactivities, respectively whereas others displayed no transfection capabilities. Note that among all these LNPs formed of triazole-containing ionizable lipids, **Cp1-4** consisting of dimethylamine was the most effective, even better than **SM-102**.

It has been reported that the proton sponge effect originates from a large number of weakly conjugated bases (which have buffer capacity in the pH range of 4.0–6.0), resulting in the absorption of protons in acidic organelle and the accumulation of osmotic pressure through organelle membranes. This osmotic pressure causes the expansion and rupture of the acidic endosomes and releases the trapped material into the cytoplasm [21].

Tertiary amines have a strong proton absorption capacity in the inner body (pH, 5–6) and lysosomes (pH, 4–5), leading to rapid osmotic expansion and mRNA release [22]. The high transfection efficiencies of **Cp1-4**, **Cp1-5**, **Cp1-6** and **Cp1-7** may be related to this.

These results thus show that the triazole linker in ionizable lipids could allow LNP formation for mRNA delivery. The variable delivery capacities suggest that minor chemical structure alterations to the triazole can also significantly impact the outcomes, which has often been observed during lipid derivatization [23]. However, these results may guide the structural design of ionizable lipid libraries in the future, and therefore, it is advantageous to include one or more tertiary amines at the end of the head structure of the triazole linkers.

## 3. Materials and Methods

### 3.1. General

All chemicals and solvents were purchased from Energy-Chemical or Bidepharm. The Quant-iT^TM^ RiboGreen^TM^ RNA Assay Kit (invitrogen) was purchased from Thermo Fisher Scientific. The Luciferase Reporter Gene Assay Kit was purchased from Yeasen Biotech. The Cell Counting Kit-8 (CCK-8) kits were purchased from Coolaber. The liposome supplement was purchased from Avito (Shanghai) Pharmaceutical Technology Co., Ltd. Luciferase mRNA was provided by Vazyme. LC/MS was performed on a LC MS-2020 resolution Mass Spectrometer, and ^1^H-NMR was performed on a Bruker AVANCE III-400 400 MHz. The particle size and zeta-potential were measured using a BrookHaven 90plus PALS dynamic light diffuser. Luciferase signals were measured using a TECAN Spark 10M multifunctional enzyme spectrometer. A LiposoFast-Basic LF-1 liposome preparation extruder was used for liposome preparation, and Kylin-Bell Lab Instruments ZD-9550 were used for dialysis.

### 3.2. LNP Formulation

The lipid composition (50% ionizable lipids, 38.5% cholesterol, 10% dsc and 1.5% DMG-PEG2000) was dissolved in 250 μL of anhydrous ethanol, and 20mg of luciferase mRNA was dissolved in 750 μL of 50 mM sodium citrate (pH = 3.0). The ratio of ionizable lipids to mRNA was 20:1 (*m*/*m*). After the lipid component was fully mixed with the mRNA component, LNPs were prepared using the extrusion mechanism of liposome. The liposome extruder used a polycarbonate membrane with a pore size of 100 nm, and each sample was extruded 21 times. The prepared LNPs were put into dialysis bags (interception molecular weight 3500) and dialysis was conducted in 1 × PBS (pH = 7.4) for 18 h. The LNPs were collected from the dialysis bags, filtered through a 0.22 μm syringe filter and stored at 4 °C.

### 3.3. LNP Size Distribution and Zeta Potential

LNPs were equilibrated at room temperature and diluted in water at a ratio of 1:200 for size and zeta potential measurements. Size distribution and zeta potential were measured using a BrookHaven 90plus PALS dynamic light Scatterer.

### 3.4. mRNA Concentration and Encapsulation Efficiency

The encapsulation rate of LNPs was measured using the RiboGreen fluorescent dye kit (Invitrogen). Its principle is that RiboGreen is a kind of super-sensitive fluorescent nucleic acid dye. RiboGreen fluorescent dye has almost no fluorescent activity in a solution state. When RiboGreen is combined with RNA, its fluorescence is turned on and its activity can be increased by approximately 1000 times. A total of 50 μL of LNP samples were transferred to the centrifuge tube and diluted to 350 μL with 1 × TE buffer solution. The 50 μL dilution of LNPs samples were added into the 96-well plate. A total of 2 μL (100:1 *v*/*v*) of Triton X-100 was added, mixed, and incubated at room temperature for 10 min. The RiboGreen reagent was then added to each well, and the fluorescence intensity was measured using a Tecan Spark 10M plate reader (ex, 480 nm; em, 525 nm). If the fluorescence signal value of the sample lysed without Triton X-100 represents the free mRNA content, and the fluorescence signal value of the sample lysed with Triton X-100 represents the total mRNA content, then the encapsulation rate is the ratio of the total mRNA content minus the free mRNA content to the total mRNA content. The raw fluorescence values were subtracted from the background fluorescence, and the total RNA concentration in the LNP formulation was determined according to the standard curve.

(1) $$Encapsulation\;Efficiency=\frac{total\;mRNA\;content-free\;mRNA\;content}{total\;mRNA\;content}\times 100\%$$

### 3.5. Cell Transfection and Luciferase Expression

HeLa cells were cultured in a DMEM high sugar medium at 37 ℃ in an incubator with 5% CO_2_. The cells were then seeded into 96-well plates, with a density of 15,000 cells/well. After 24 h, 1 μg of mRNA or LNPs with 1 μg of mRNA were added into each well. The cells were further cultured for 24 h. After lysis using the cell lysis buffer, the cell lysate was transferred into 96-well plates. A total of 20 μL of luciferase substrate was then added, and the bioluminescence signals were further measured. The luciferase intensity data were normalized and compared.

## 4. Conclusions

In summary, we have used the biorthogonal CuAAC to synthesize a library of triazole-containing ionizable lipids **Cp1-n**. A cell viability assay demonstrated that the introduction of triazole into ionizable lipid structures was biocompatible. **Cp1-n** were then further applied in order to prepare LNPs for luciferase mRNA delivery. We showed that LNPs could be easily formed using these triazole-containing ionizable lipids, which had an average size of ~100 nm and a surface potential of +15 mV. Moreover, LNPs formed of **Cp1-n** could efficiently package luciferase mRNA, with encapsulation rates of ~85%. The mRNA delivery efficacy of **Cp1-4**-based LNPs was found to be 1.32 times higher than that of **SM-102**-based LNPs.

Overall, our work provides a facile chemical approach to rapidly prepare triazole-containing ionizable lipids, which are also suitable for LNP preparation and mRNA delivery. Similar bioorthgonal chemistries could also be employed to quickly diversify the ionizable lipid libraries with distinct chemical structures and properties. In the future, we will continue to explore chemical strategies to expand the library of triazole-containing ionizable lipids and elucidate their structure–activity relationships for mRNA delivery.

## Figures and Tables

**Table 1:** Zeta potentials of LNPs formed of **SM-102** or **Cp1-n**.

**Lipid**	**SM-102**	**Cp1-1**	**Cp1-2**	**Cp1-3**	**Cp1-4**	**Cp1-5**	**Cp1-6**	**Cp1-7**
Zeta Potential of LNPs (mV)	+ 15.77 ± 1.25	+ 16.68 ± 1.88	+ 14.90 ± 0.44	+ 10.64 ± 0.67	+ 14.63 ± 0.68	+ 15.28 ± 1.24	+ 13.45 ± 2.58	+ 11.79 ± 0.71
**Lipid**	**Cp1-8**	**Cp1-9**	**Cp1-10**	**Cp1-11**	**Cp1-12**	**Cp1-13**	**Cp1-14**	
Zeta Potential of LNPs (mV)	+ 18.71 ± 1.54	+ 25.41 ± 0.90	+ 19.26 ± 2.06	+ 11.46 ± 2.40	+ 25.04 ± 0.86	+ 8.64 ± 0.97	+ 13.45 ± 1.69	

**Table 2:** The luciferase mRNA encapsulation rates of LNPs formed of different lipids.

Lipid	Encapsulation Rate (%)	Lipid	Encapsulation Rate (%)
**SM-102**	89.98 ± 0.78	**Cp1-8**	88.86 ± 1.53
**Cp1-1**	88.20 ± 4.61	**Cp1-9**	88.01 ± 3.44
**Cp1-2**	91.64 ± 4.59	**Cp1-10**	84.52 ± 6.04
**Cp1-3**	89.28 ± 2.42	**Cp1-11**	84.10 ± 1.00
**Cp1-4**	89.55 ± 1.92	**Cp1-12**	88.24 ± 3.83
**Cp1-5**	91.53 ± 0.68	**Cp1-13**	85.87 ± 4.44
**Cp1-6**	91.92 ± 3.09	**Cp1-14**	89.28 ± 2.40
**Cp1-7**	91.53 ± 3.40		

## Data Availability

The data presented in this study are available on request from the corresponding author.

## Supplementary Materials

The following supporting information can be downloaded at: https://www.mdpi.com/article/10.3390/molecules28104046/s1, Chemical synthesis and characterization. Figure S1: cytotoxicity of **Cp1-n**. Table S1: The ratio of reactant. Table S2: The yield of CuAAC reaction.

## Sample Availability

Samples of the compounds **Cp1-n** and **SM-102** are available from the authors.
