# Supplementary material for: Ionizable Lipids with Triazole Moiety from Click Reaction for LNP-Based mRNA Delivery
Source: Molecules. 2023 May 12;28(10):4046. doi: 10.3390/molecules28104046 (PMC10223118; doi:10.3390/molecules28104046)

Supporting Information

Ionizable Lipids with Triazole Moiety from Click Reactions for LNP-based mRNA Delivery

Yixiang Wang [1], Xiao Si [1], Yi Feng [1], Dan Feng [2], Xiaoyu Xu [2,*] and Yan Zhang [1,*]

[1] School of Chemistry and Chemical Engineering, Chemistry and Biomedicine Innovation Center (ChemBIC), Jiangsu Key Laboratory of Advanced Organic Materials, State Key Laboratory of Analytical Chemistry for Life Sciences; Nanjing University, Nanjing 210023,China

[2] Nanjing Vazyme Biotechnology Company, Nanjing 210034, China

* Correspondence: xuxiaoyu@vazyme.com (X.X.); njuzy@nju.edu.cn (Y.Z.)

Synthesis of B_1

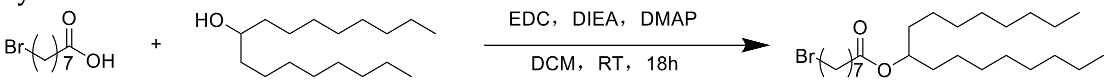

R1 R2 B1 58.5%

Bromooctanoic acid (R1, 10.0008 g，0.0448 mol), heptadecan-9-ol (R2, 12.6458 g，0.0493 mol), EDCI (12.8822 g，0.0672 mol), DIEA (14.4890 g，0.1121 mol), and DMAP (0.8214 g，0.0067 mol) were dissolved in DCM. The mixture was stirred for 18 h at room temperature. After the solvent was removed, the residue was purified by column chromatography with gradient elution from 100% Petroleum ether (PE) to PE/EA (100/1, v/v) to give **B_1**. The yield was 58.5%.

^{1}H NMR (400 MHz, Chloroform-d) δ 4.87 (p, J=6.3Hz, 1H), 3.40 (t, J=6.8Hz, 2H), 2.28 (t, J=7.4Hz, 2H), 1.85 (p, J=6.9Hz, 2H), 1.63 (dddd, J=12.3, 7.5, 4.7, 2.2Hz, 2H), 1.54-1.40 (m, 6H), 1.35-1.21 (m, 28H), 0.96-0.81 (m, 6H).

Synthesis of SM-102

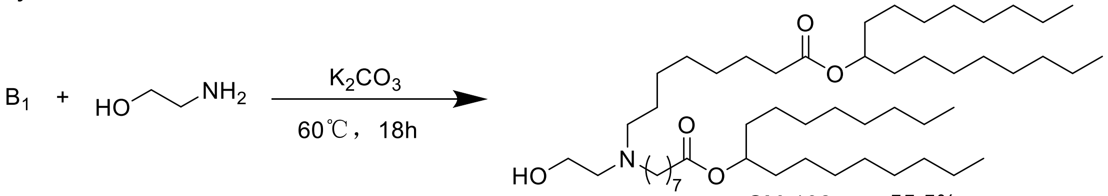

SM-102 55.5%

B_1 (8.0006 g，0.0173 mol) was reacted with 2-aminoethane-1-ol (30 ml) in ethanol at 60 ℃ for 18 h. After the reaction was completed, the residue was purified by column chromatography with gradient elution from 100% PE to PE/EA (5/1, v/v) to give **SM-102**. The yield was 55.5%.

^{1}H NMR (400 MHz, Chloroform-d) δ 4.86 (p, J=6.3Hz, 1H), 4.17-4.03 (m, 4H), 3.61 (t, J=5.3Hz, 2H), 2.67 (t, J=5.3Hz, 2H), 2.60-2.50 (m, 4H), 2.29 (dt, J=10.6, 7.4Hz, 4H), 1.62 (dq, J=9.9, 7.2, 6.7Hz, 6H), 1.55-1.44 (m, 8H), 1.35-1.22 (m, 53H), 0.99-0.84 (m, 9H). LC-MS: m/z 710.80 $[M+H]^+$ $C_{44}H_{87}NO_5$ (cacld. 710.18).

Synthesis of Az1

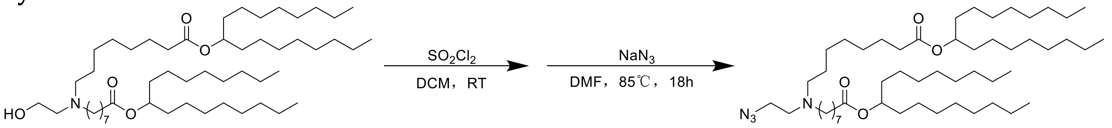

SM-102 Az1 76.5%

SM-102 (4.9200 g，0.0060 mol) was added into a round-bottomed flask and dissolved in DCM. SO2Cl2(2.4235 g，0.0180 mol) was dripped at room temperature while stirring. Then the reaction was stirred at room temperature for 10 min. After the reaction was completed, crude product **Cl-S1** was extracted. The crude product of **Cl-S1** was dissolved directly by DMF, and NaN_3 (0.7782 g，0.0120 mol) was added. The mixture was stired at room temperature for 10 min. Then the reaction was kept at 85 ℃ for 18 h. After the reaction was completed, the residue was purified by column chromatography with gradient elution from 100% PE to PE/EA (50/1, v/v) to give the **Az1**. The yield was 76.5%.

^{1}H NMR (400 MHz, Chloroform-d) δ 4.86 (p, J=6.3Hz, 1H), 4.06 (t, J=6.7Hz, 2H), 3.24 (t, J=6.2Hz, 2H), 2.63 (t, J=6.2Hz, 2H), 2.54-2.37 (m, 4H), 2.29 (dt, J=9.3, 7.5Hz, 4H), 1.69-1.57 (m, 8H), 1.55-1.39 (m, 9H), 1.37-1.21 (m, 49H), 0.88 (td, J=6.9, 1.6Hz, 9H). LC-MS: m/z 735.60 $[M+H]^+$ $C_{44}H_{86}N_4O_4$ (cacld. 735.20).

Synthesis of ionizable lipid Cp1-n

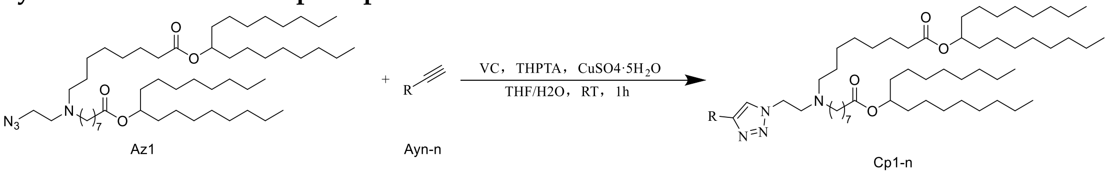

Az1 Ayn-n Cp1-n

Firstly, **Az1**, VC, THPTA, CuSO4 and terminal alkyne were prepared as shown in Table 3. The solvent was a mixture of THF: H_2O: DMSO (4:1:0.05). The reaction was stirred at room temperature for 1 h, and the reaction was monitored by TLC. After the reaction was completed, the reaction solution was evaporated, redissolved in EA, and washed with saturated NaCl for 5 times to obtain **Cp1-n**. The yield was 85.3%-90.6%.

	**Az1**	VC	THPTA	$CuSO_4{\cdot}5H_2O$
M	735.20	176.13	434.25	249.68
eq	1	1	0.1	1
m/mg	100.0	23.9	5.9	34.0

	**Ayn-1**	**Ayn -2**	**Ayn -3**	**Ayn -4**	**Ayn -5**
M	84.12	84.12	84.12	83.13	111.19
eq			1.2		
m/mg	13.7	13.7	13.7	13.6	18.1

	**Ayn -6**	**Ayn -7**	**Ayn -8**	**Ayn -9**	**Ayn -10**
M	109.17	138.21	69.11	68.12	82.15
eq			1.2		
m/mg	17.8	22.6	11.3	11.1	13.4

	**Ayn -11**	**Ayn -12**	**Ayn -13**	**Ayn-14**
M	66.10	140.18	128.17	102.14
eq			1.2	
m/mg	10.8	22.9	20.9	16.7

Table S1. The ratio of reactant

**Lipid**	**yield**	**Lipid**	**Yield**
**Cp1-1**	88.43	**Cp1-8**	83.29
**Cp1-2**	79.14	**Cp1-9**	88.06
**Cp1-3**	90.30	**Cp1-10**	89.95
**Cp1-4**	86.26	**Cp1-11**	84.08
**Cp1-5**	87.22	**Cp1-12**	86.16
**Cp1-6**	91.24	**Cp1-13**	88.82
**Cp1-7**	85.73	**Cp1-14**	93.07

Table S2. The yield of CuAAC reaction

Cytotoxicity analysis of ionizable lipids

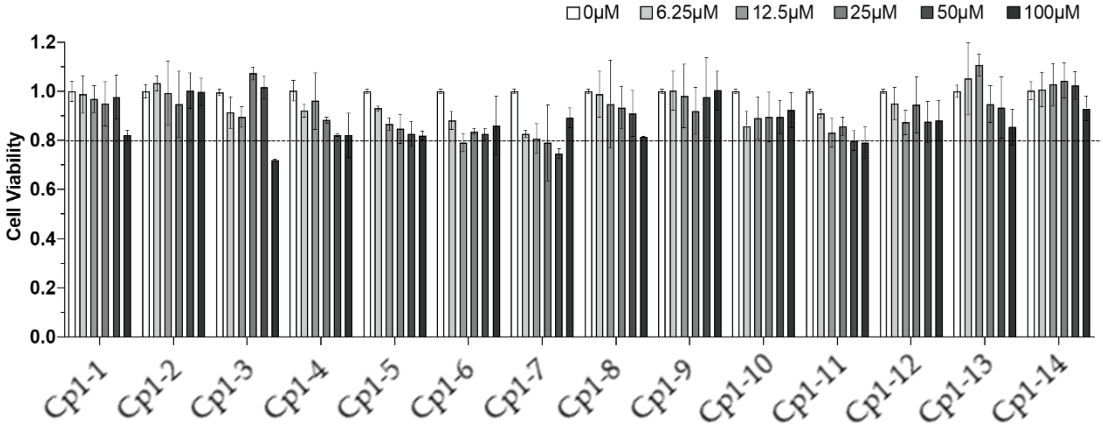

Figure S1. Cytotoxicity of Cp1-n

Mass spectrum data

Mass spectrum of SM-102

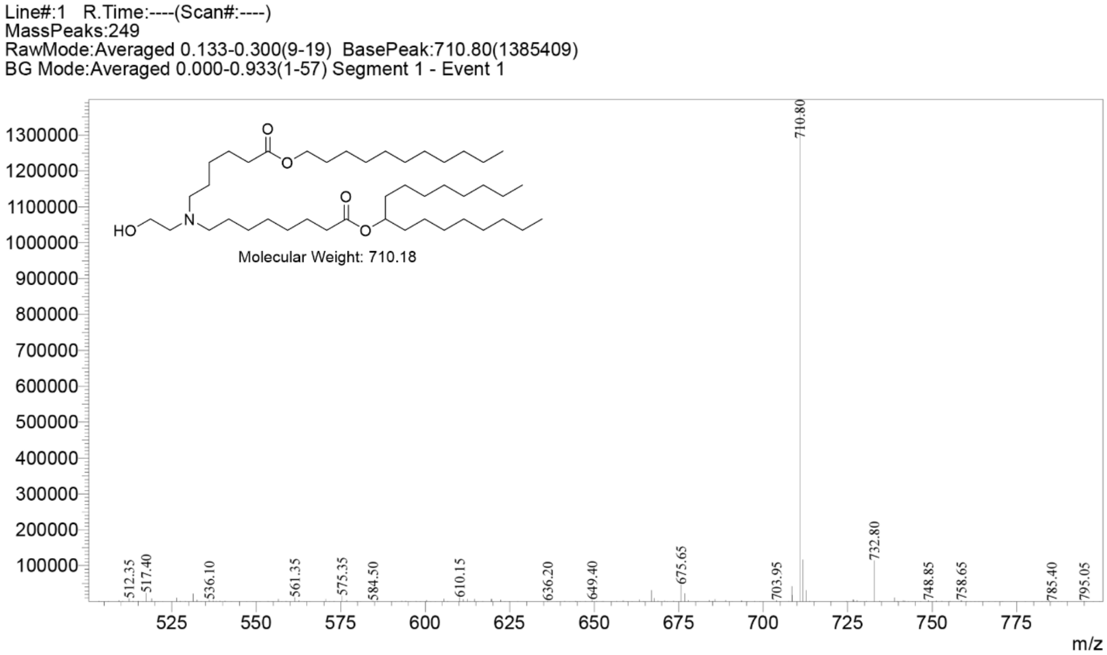

Mass spectrum of Cp1-1

Line#:1 R.Time:----(Scan#:----)
MassPeaks:779
RawMode:Averaged 0.133-0.467(9-29) BasePeak:819.95(1040206)
BG Mode:Averaged 0.000-0.933(1-57) Segment 1 - Event 1

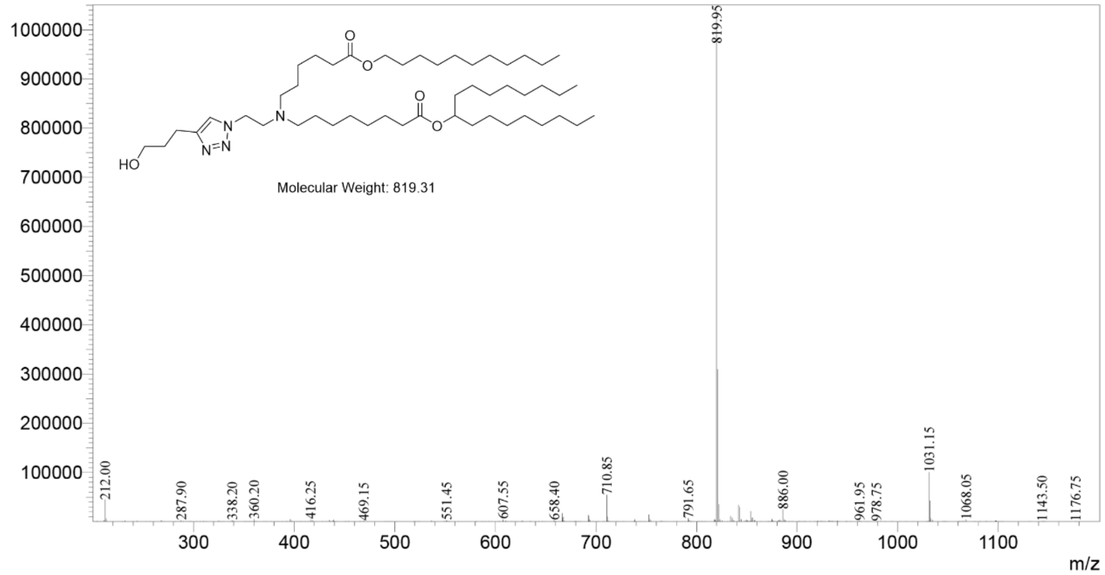

Mass spectrum of Cp1-2

Line#:1 R.Time:----(Scan#:----)
MassPeaks:535
RawMode:Averaged 0.333-0.467(21-29) BasePeak:820(4745929)
BG Mode:Averaged 0.000-0.933(1-57) Segment 1 - Event 1

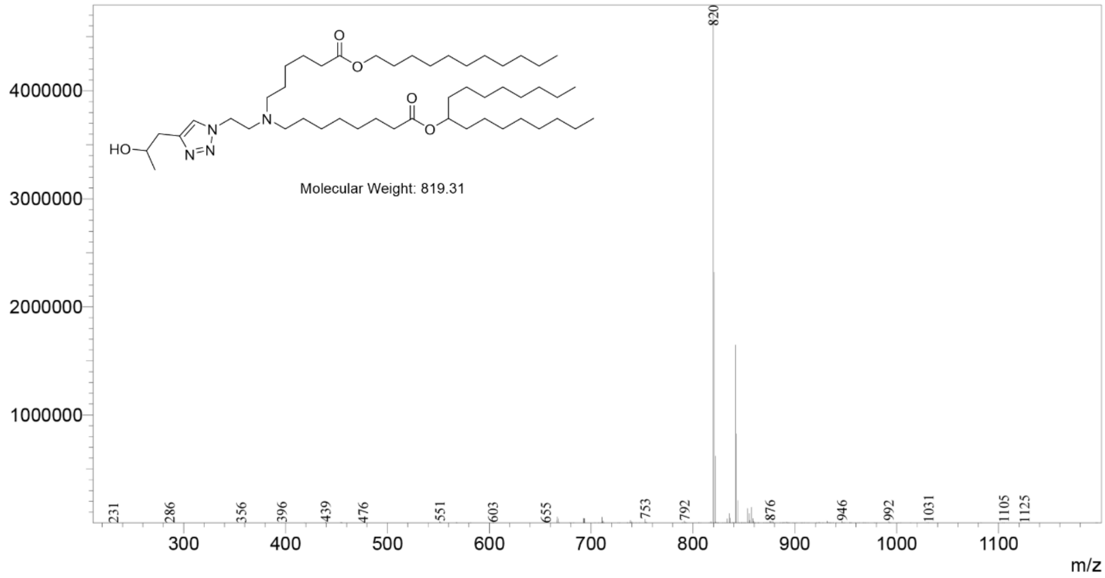

Mass spectrum of Cp1-3

Line#:1 R.Time:----(Scan#:----)
MassPeaks:480
RawMode:Averaged 0.200-0.333(13-21) BasePeak:820(3056900)
BG Mode:Averaged 0.000-0.933(1-57) Segment 1 - Event 1

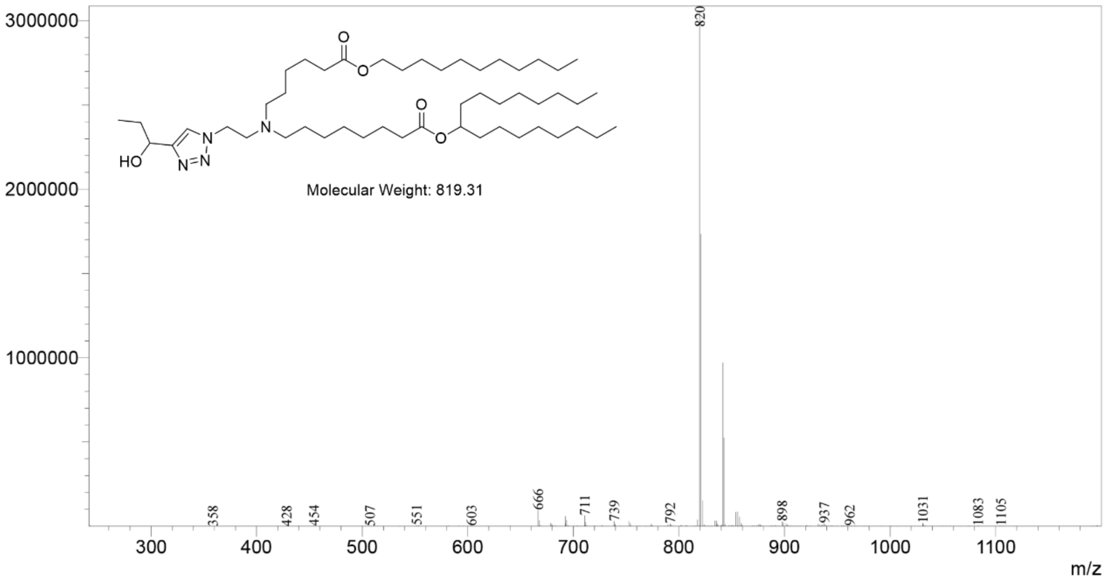

Mass spectrum of Cp1-4

Line#:1 R.Time:----(Scan#:----)
MassPeaks:387
RawMode:Averaged 0.167-0.333(11-21) BasePeak:819(10066794)
BG Mode:Averaged 0.000-0.933(1-57) Segment 1 - Event 1

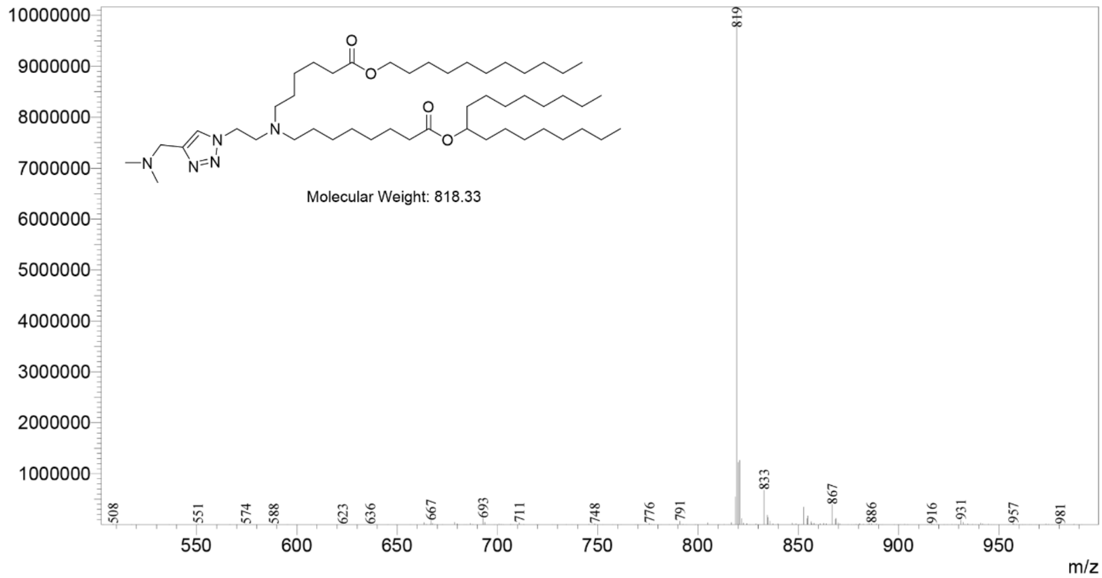

Mass spectrum of Cp1-5

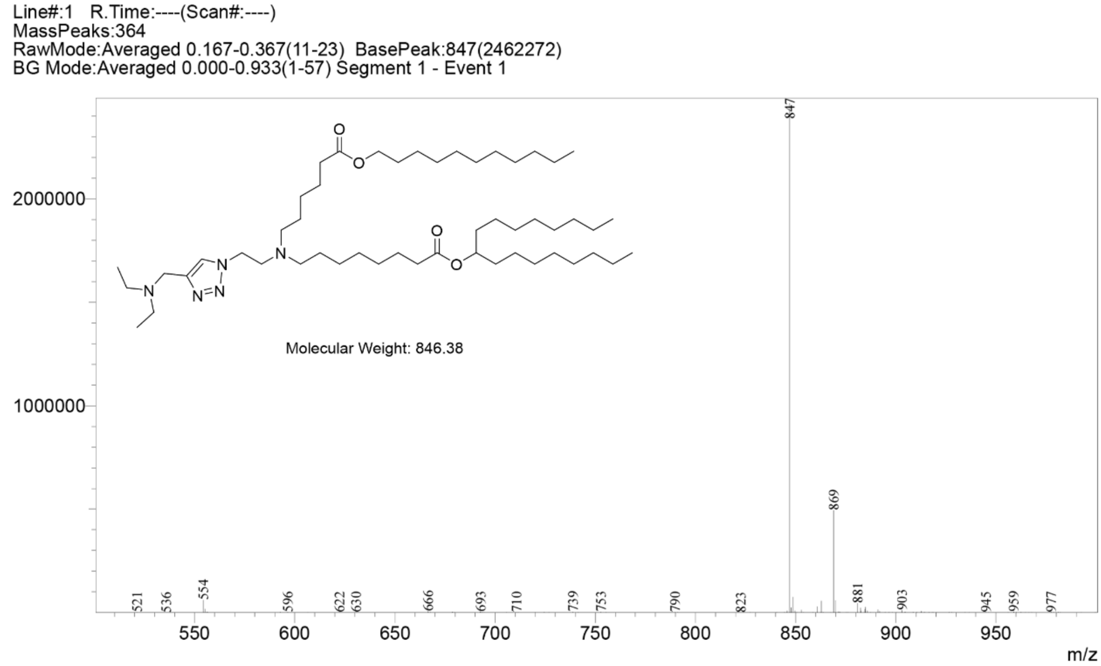

Mass spectrum of Cp1-6

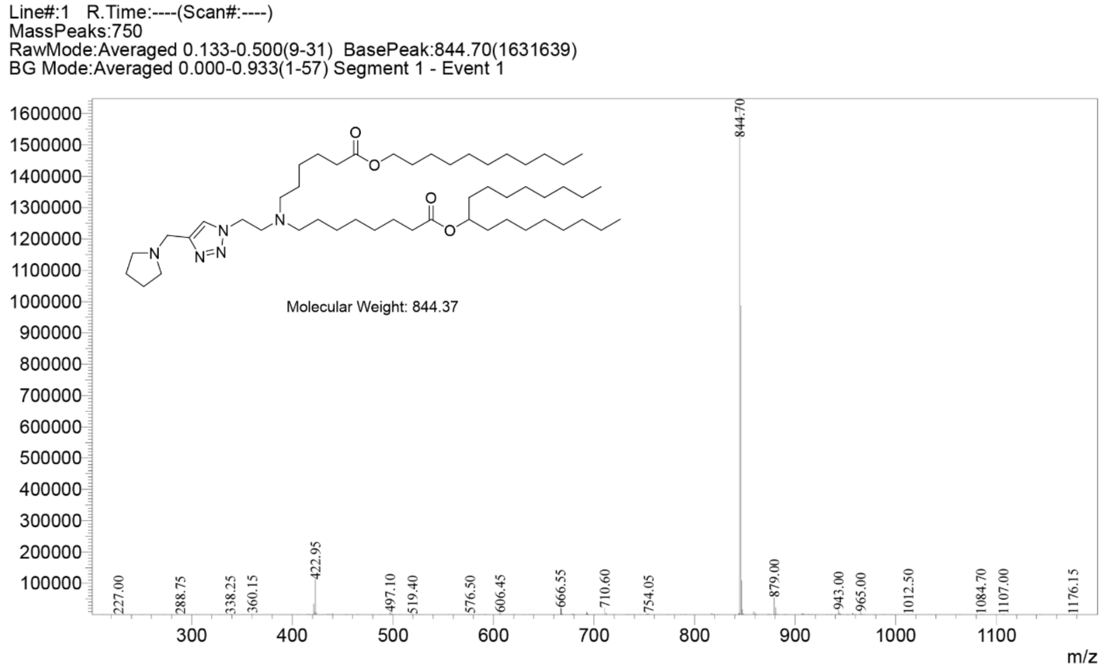

Mass spectrum of Cp1-7

Line#:1 R.Time:----(Scan#:----)
MassPeaks:499
RawMode:Averaged 0.167-0.333(11-21) BasePeak:874(15655955)
BG Mode:None Segment 1 - Event 1

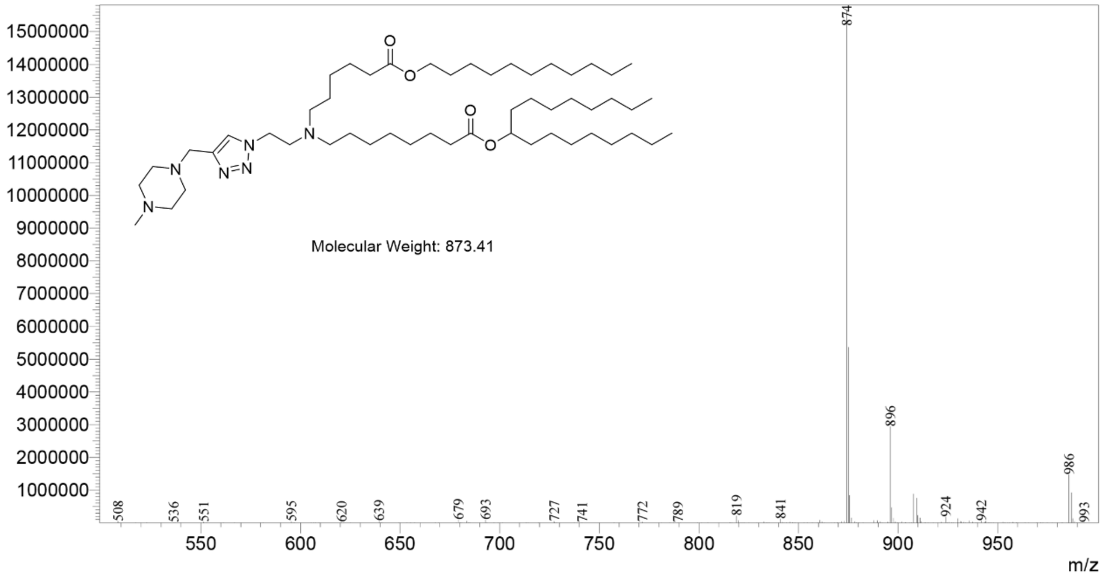

Mass spectrum of Cp1-8

Line#:1 R.Time:0.700(Scan#:43)
MassPeaks:511
RawMode:Single 0.700(43) BasePeak:805(7013060)
BG Mode:None Segment 1 - Event 1

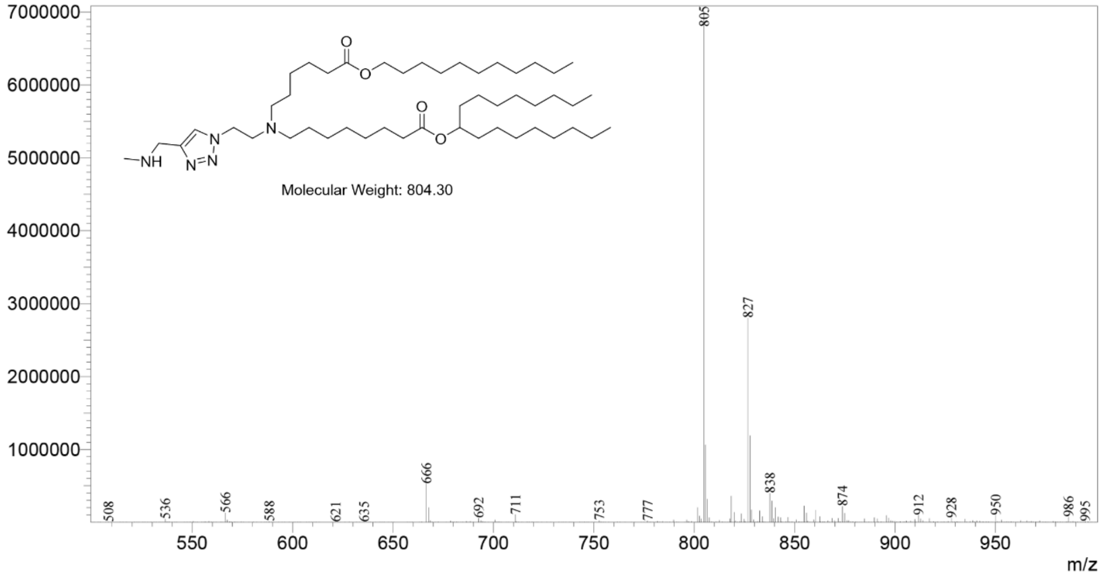

Mass spectrum of Cp1-9

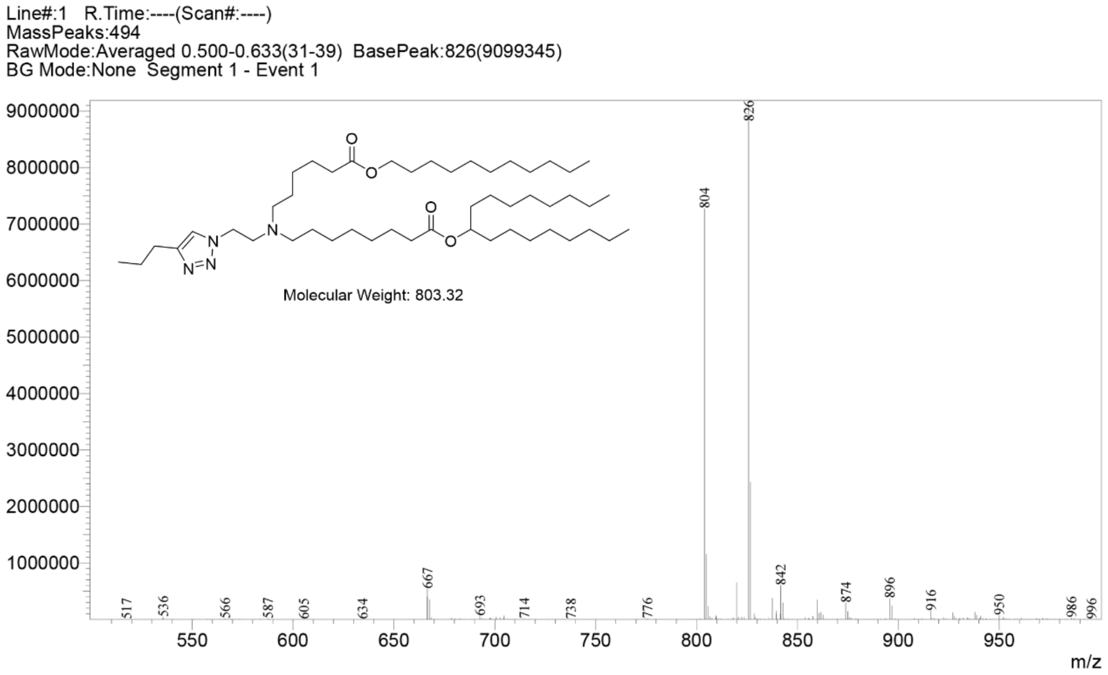

Mass spectrum of Cp1-10

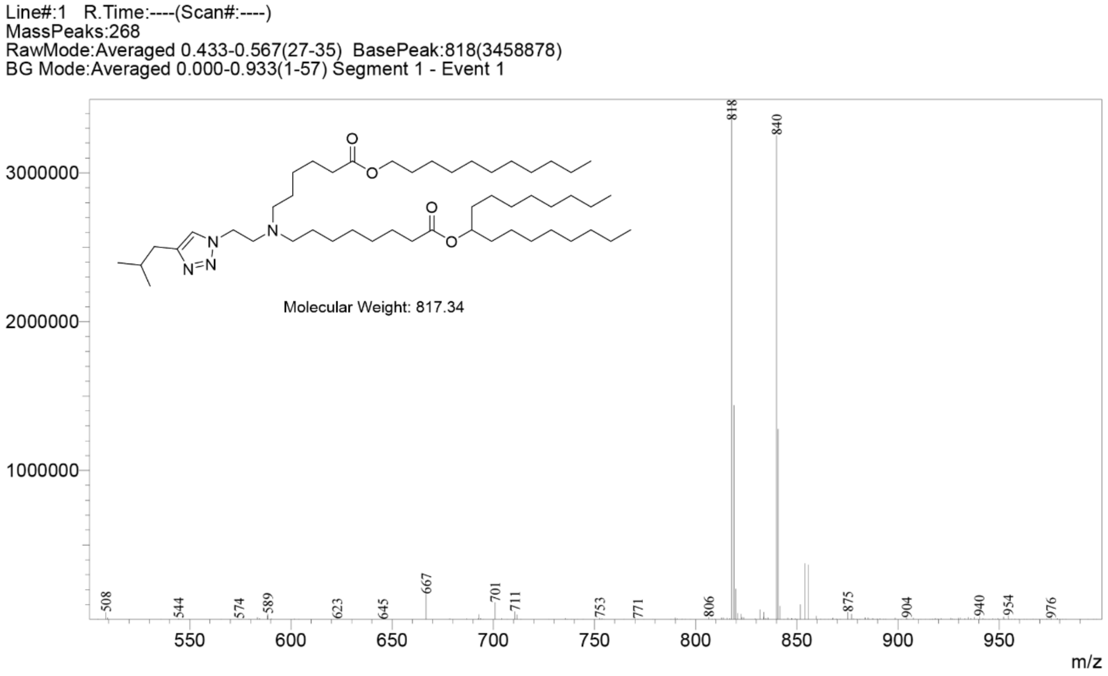

Mass spectrum of Cp1-11

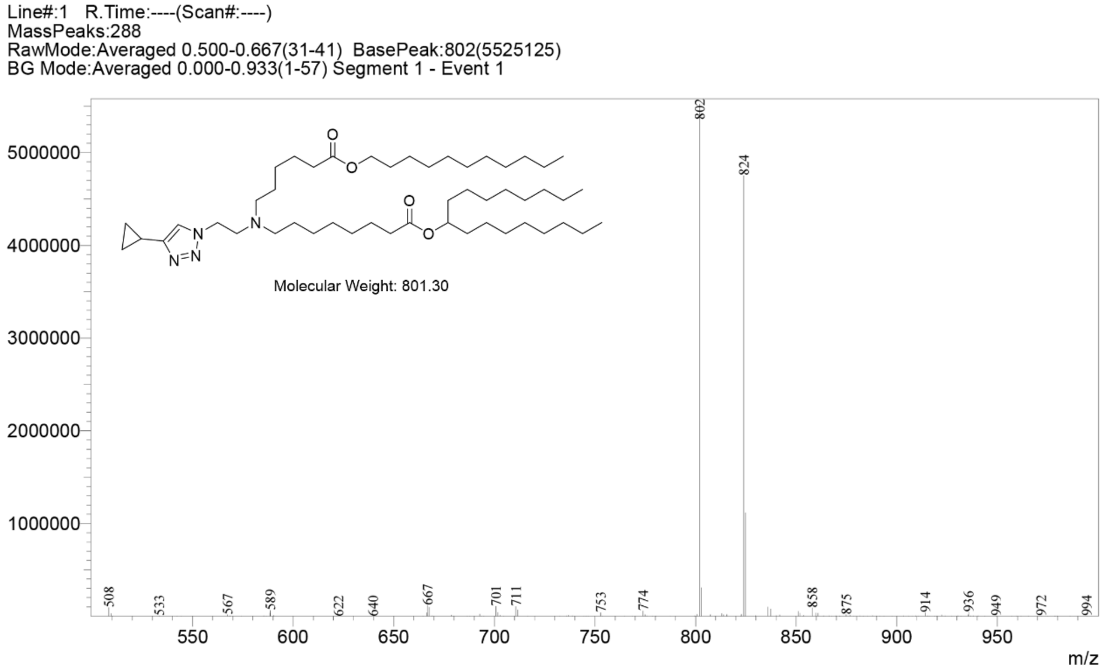

Mass spectrum of Cp1-12

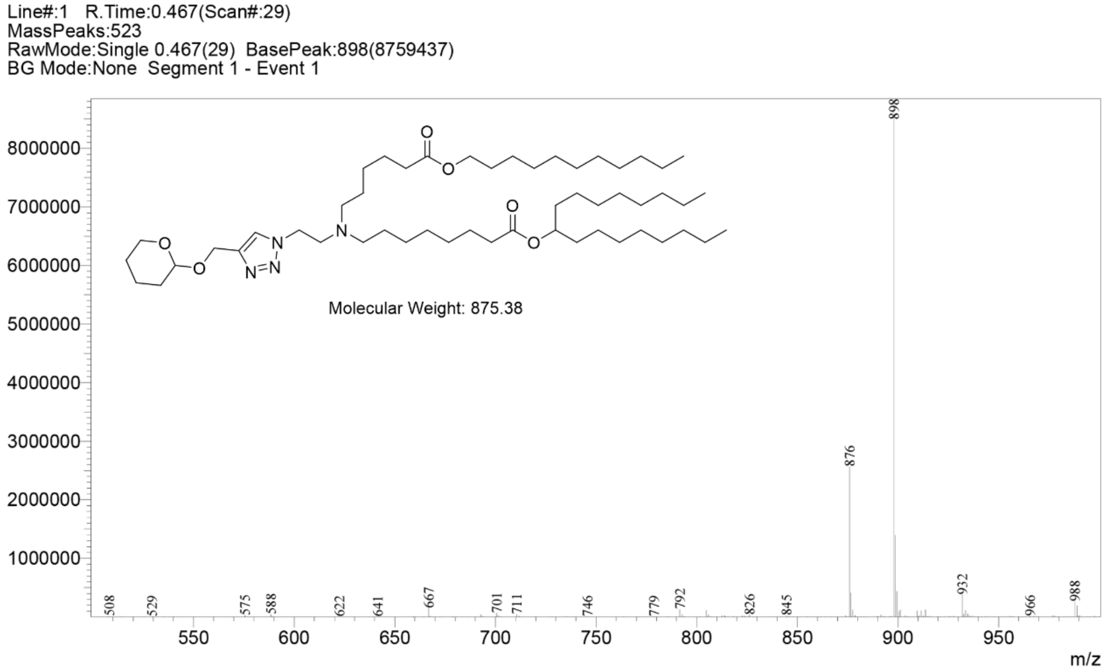

Mass spectrum of Cp1-13

Line#:1 R.Time:0.700(Scan#:43)
MassPeaks:245
RawMode:Single 0.700(43) BasePeak:864(2655817)
BG Mode:Averaged 0.433-0.900(27-55) Segment 1 - Event 1

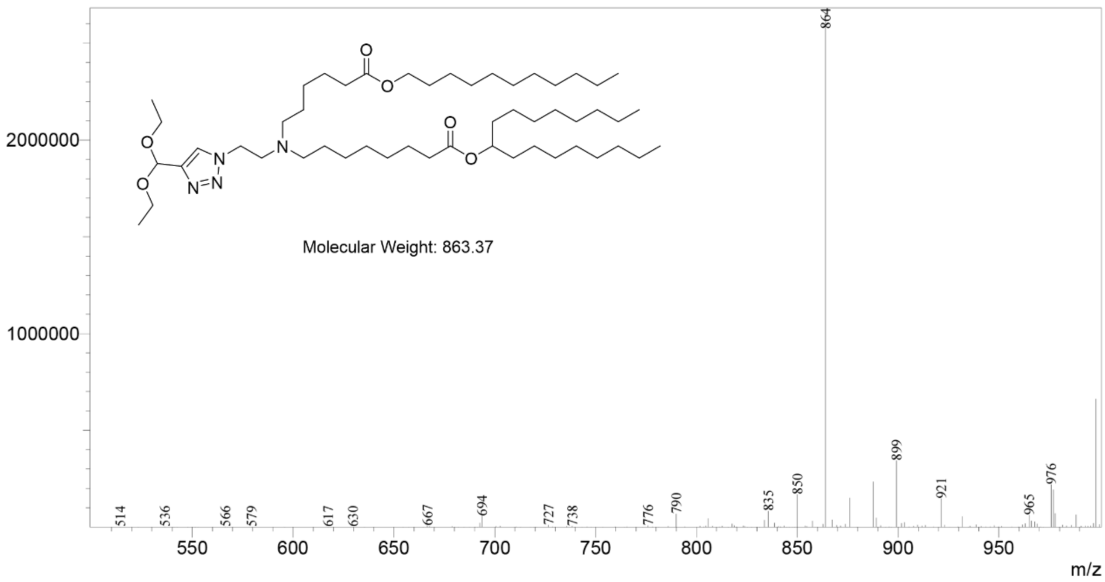

Mass spectrum of Cp1-14

Line#:1 R.Time:----(Scan#:----)
MassPeaks:272
RawMode:Averaged 0.500-0.667(31-41) BasePeak:838(3907200)
BG Mode:Averaged 0.000-0.933(1-57) Segment 1 - Event 1

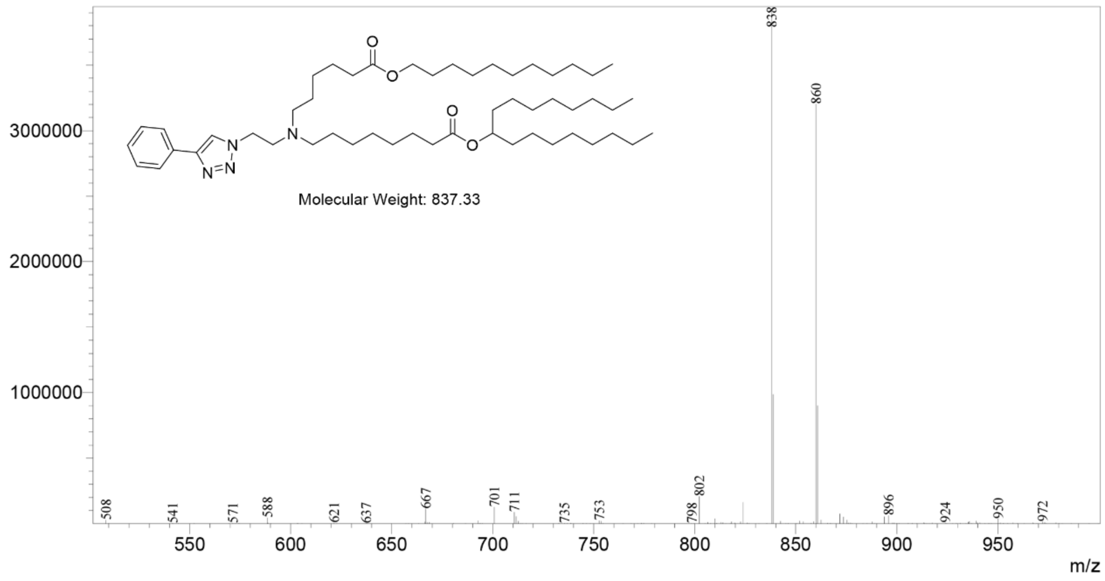

NMR Spectra

^{1}HNMR of SM-102

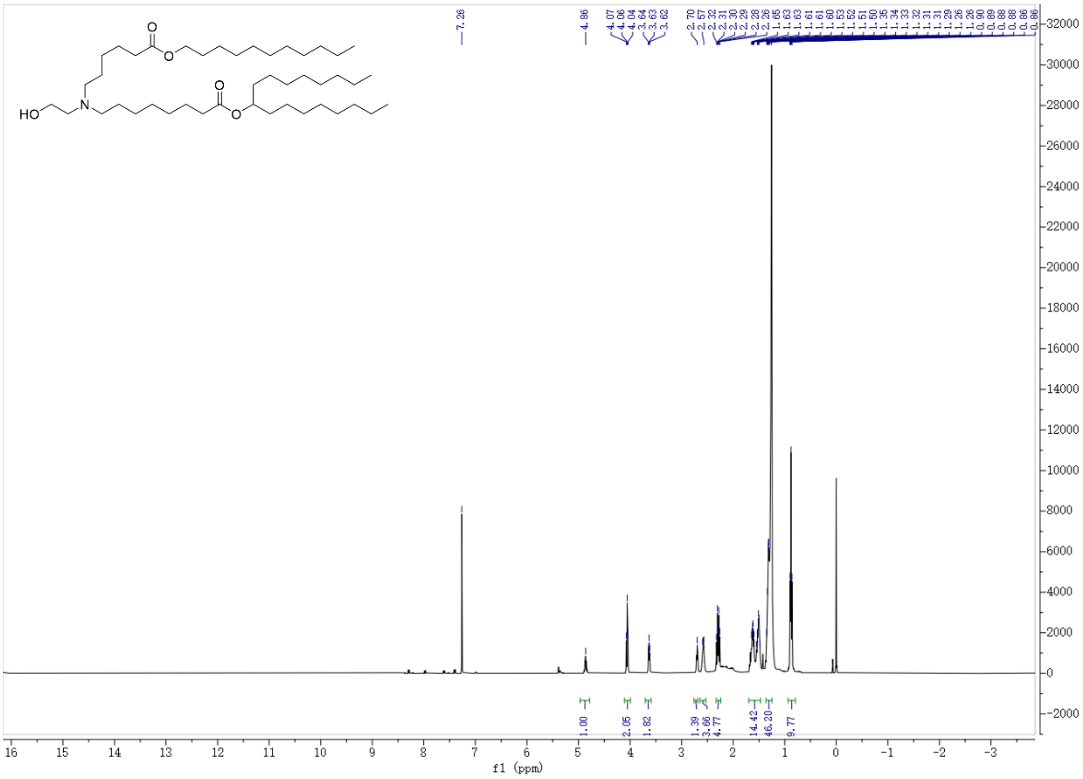

^{1}HNMR of Cp1-1

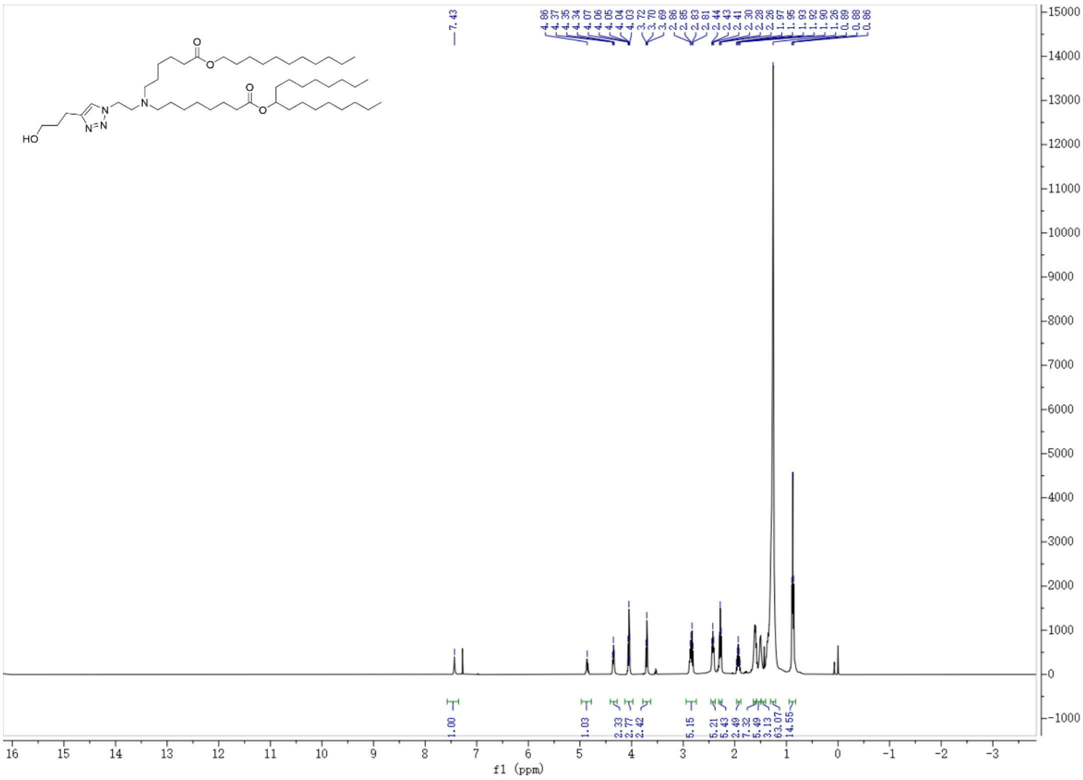

^{1}HNMR of Cp1-2

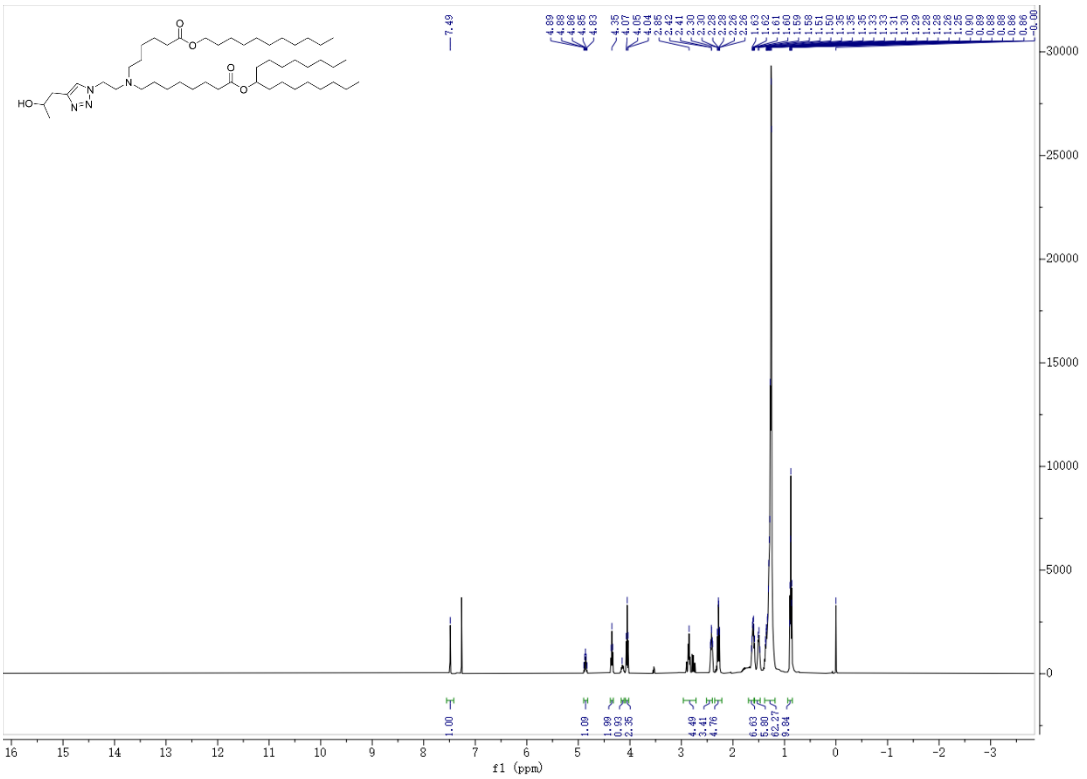

^{1}HNMR of Cp1-3

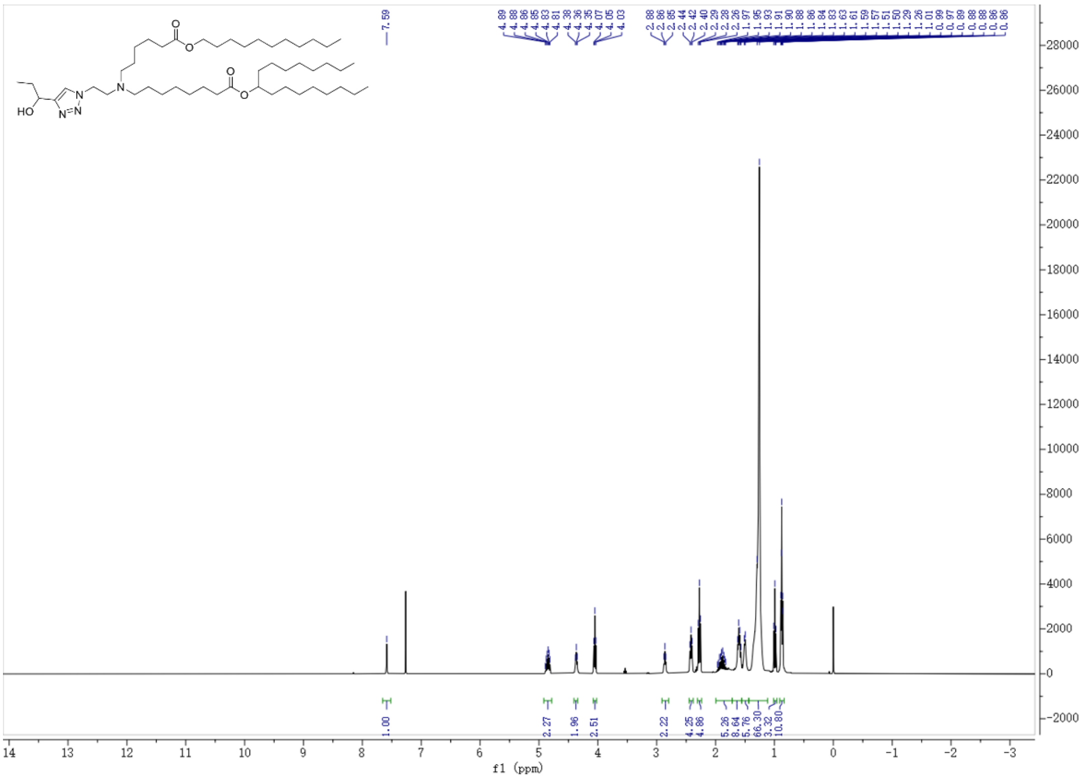

^{1}HNMR of Cp1-4

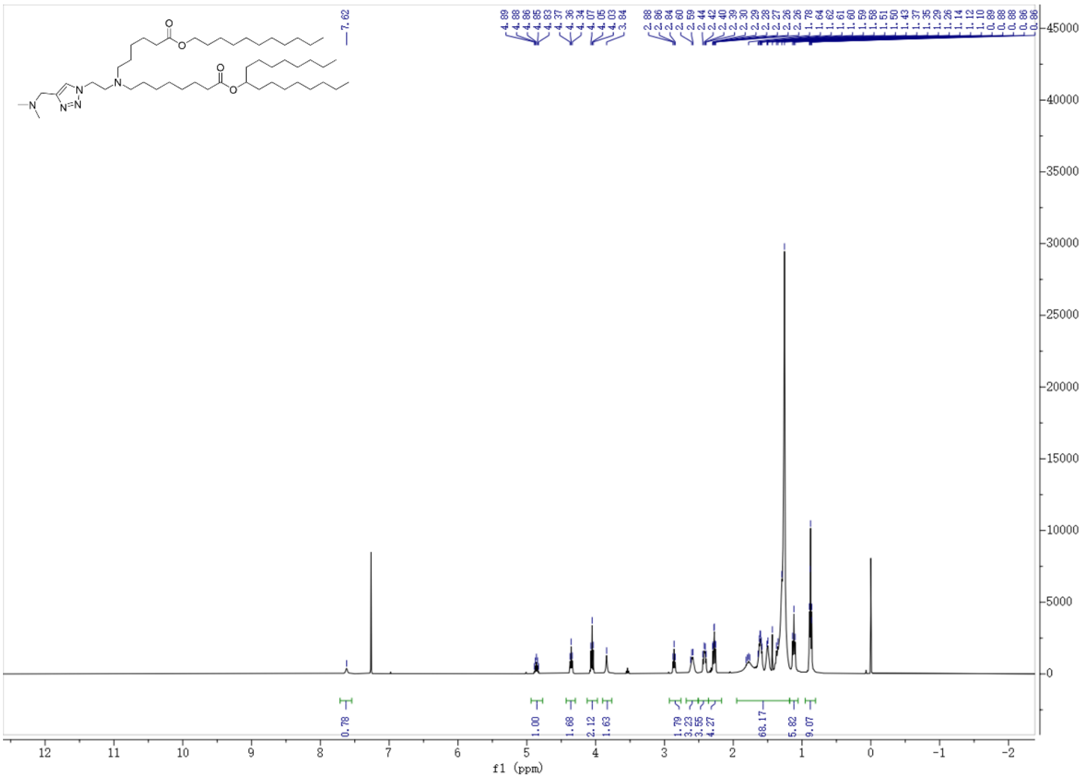

^{1}HNMR of Cp1-5

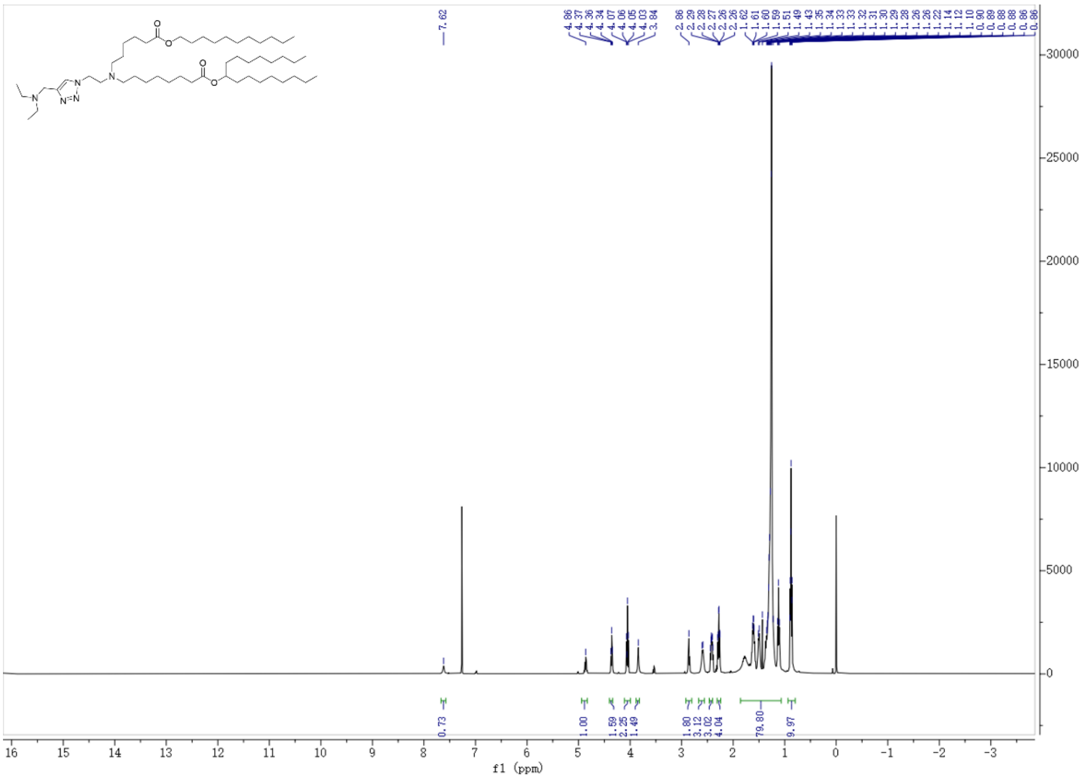

^{1}HNMR of Cp1-6

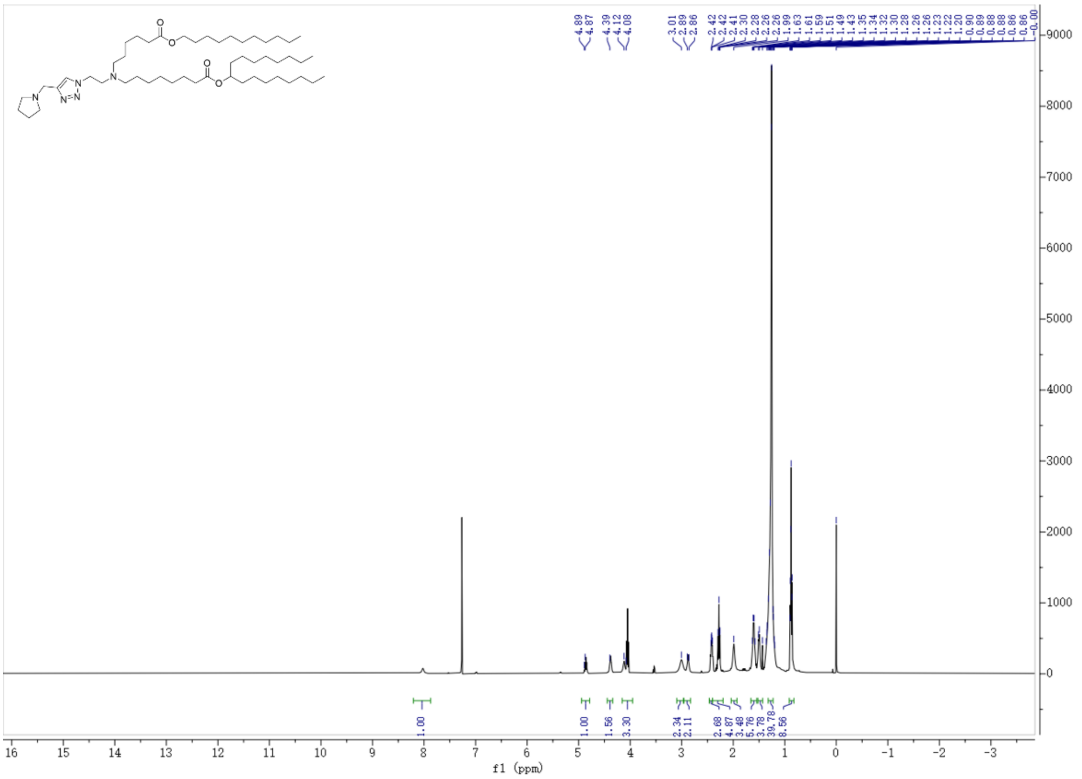

^{1}HNMR of Cp1-7

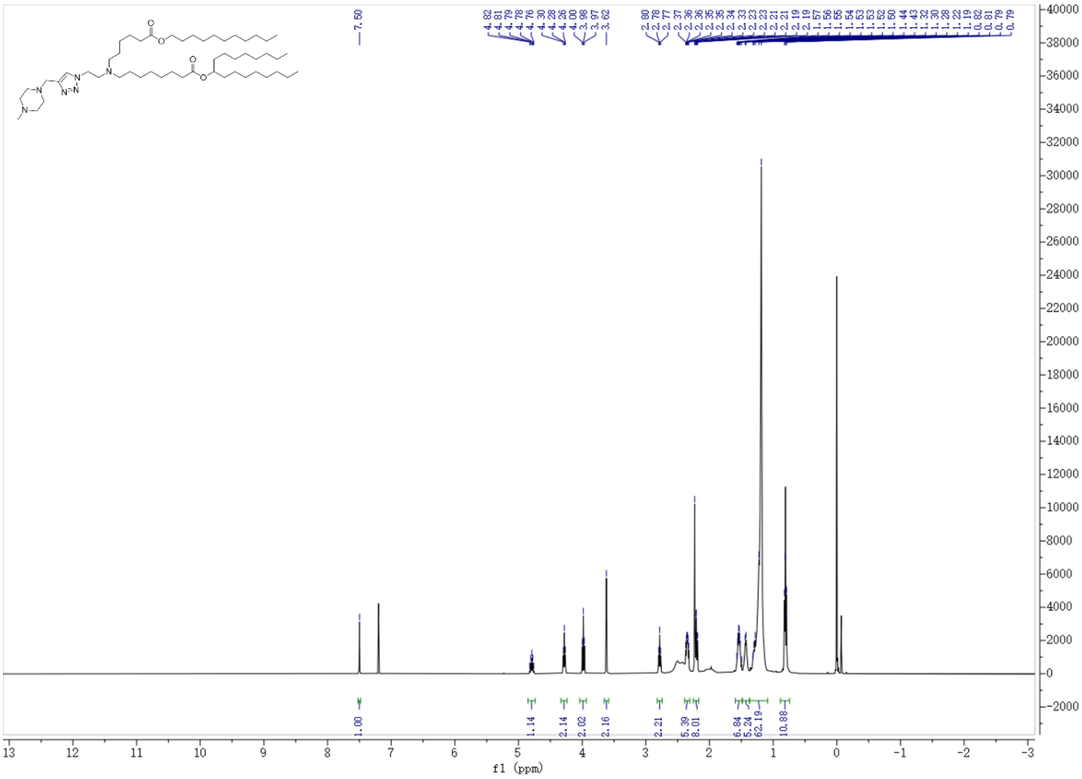

^{1}HNMR of Cp1-8

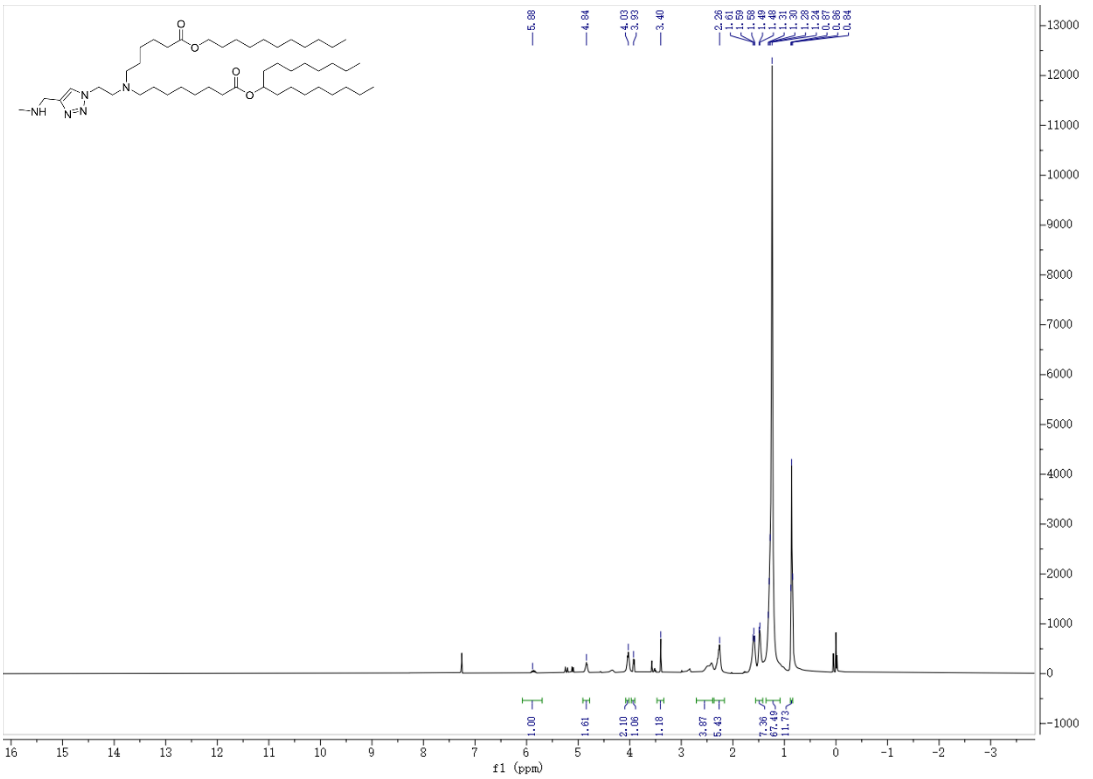

^{1}HNMR of Cp1-9

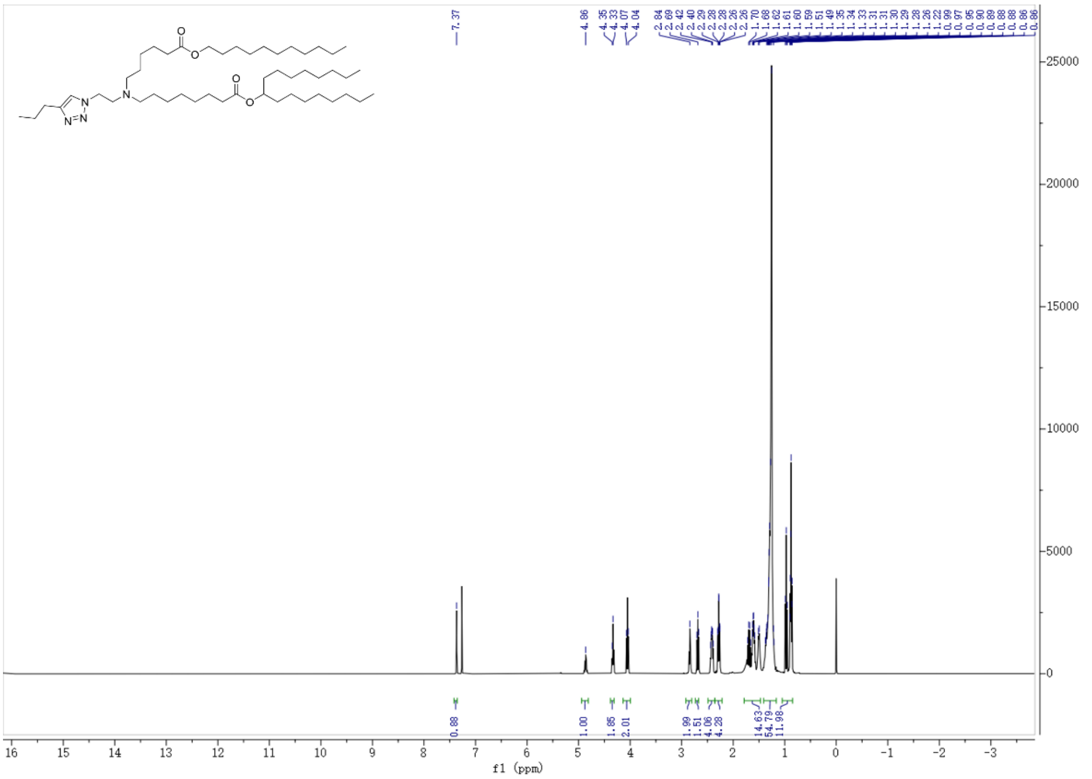

^{1}HNMR of Cp1-10

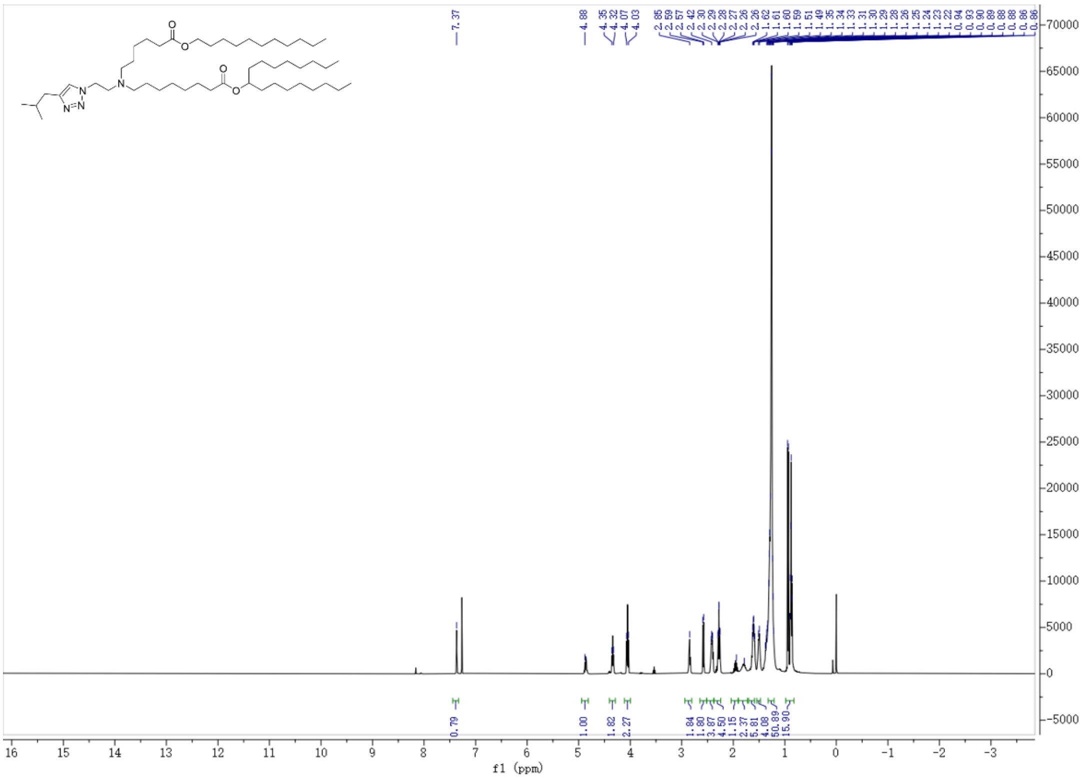

^{1}HNMR of Cp1-11

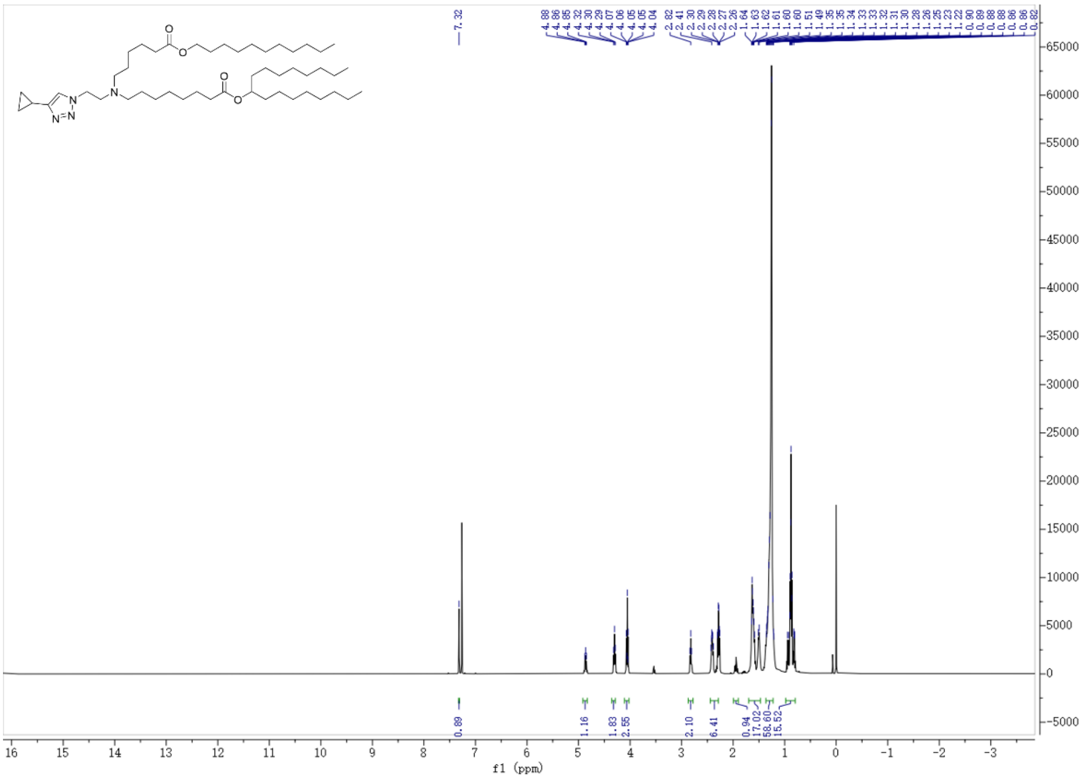

^{1}HNMR of Cp1-12

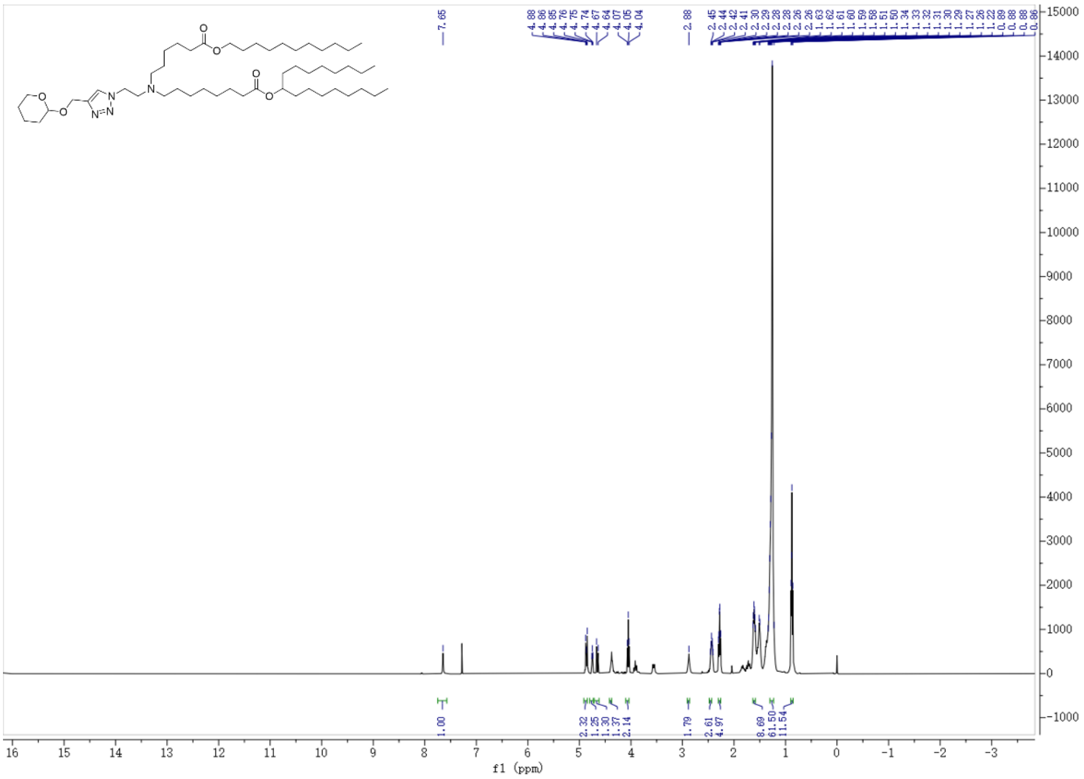

^{1}HNMR of Cp1-13

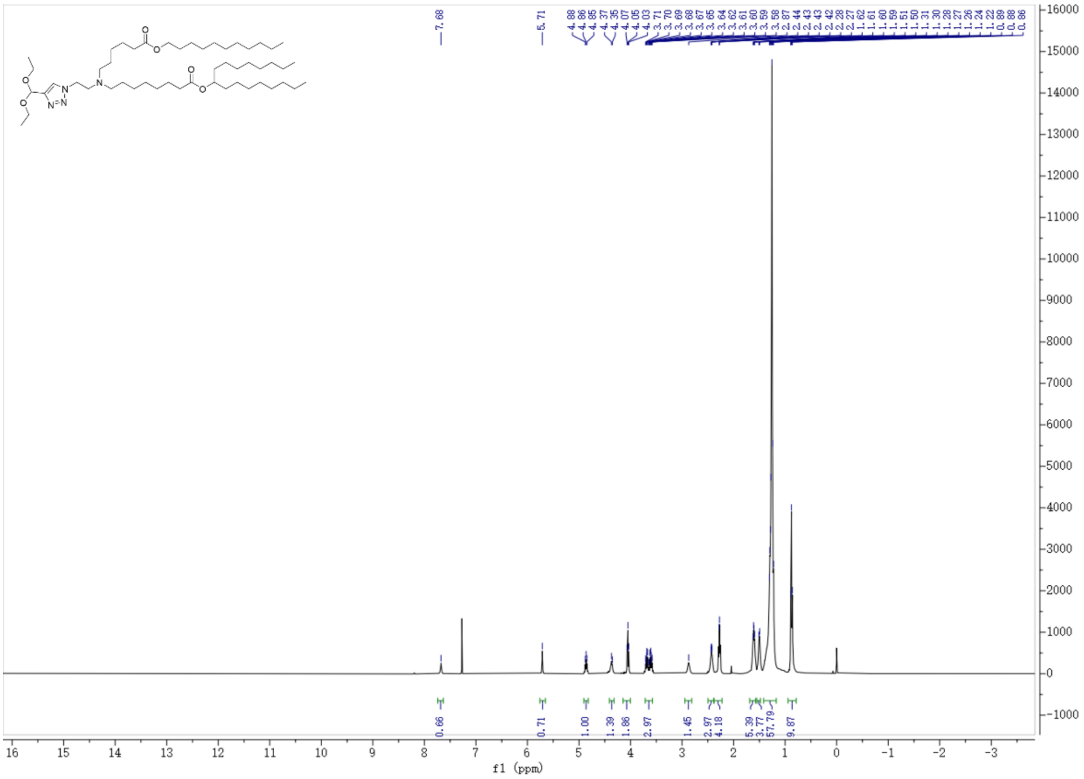

^{1}HNMR of Cp1-14

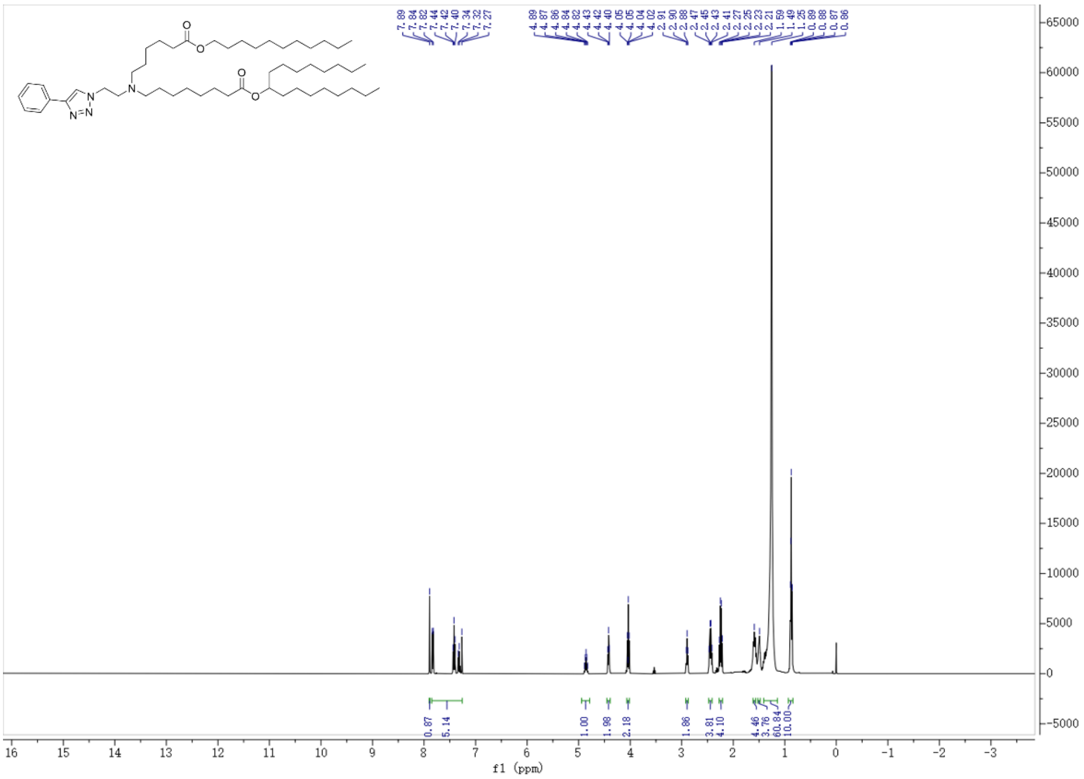

Supplement: Supplementary file 1 [file molecules-28-04046-s001.zip › molecules-2330413-supplementary.pdf]
